# Recent methodological advances in federated learning for healthcare

**DOI:** 10.1016/j.patter.2024.101006

**Published:** 2024-06-14

**Authors:** Fan Zhang, Daniel Kreuter, Yichen Chen, Sören Dittmer, Samuel Tull, Tolou Shadbahr, BloodCounts! consortium, Martijn Schut, Folkert Asselbergs, Sujoy Kar, Suthesh Sivapalaratnam, Sophie Williams, Mickey Koh, Yvonne Henskens, Bart de Wit, Umberto D’Alessandro, Bubacarr Bah, Ousman Secka, Parashkev Nachev, Rajeev Gupta, Sara Trompeter, Nancy Boeckx, Christine van Laer, Gordon A. Awandare, Kwabena Sarpong, Lucas Amenga-Etego, Mathie Leers, Mirelle Huijskens, Samuel McDermott, Willem H. Ouwehand, James Rudd, Carola-Bibiane Schӧnlieb, Nicholas Gleadall, Michael Roberts, Jacobus Preller, James H.F. Rudd, John A.D. Aston, Carola-Bibiane Schönlieb, Nicholas Gleadall, Michael Roberts

**Affiliations:** 1 Department of Applied Mathematics and Theoretical Physics, University of Cambridge, Cambridge, UK; 2 ZeTeM, University of Bremen, Bremen, Germany; 3 Research Program in Systems Oncology, Faculty of Medicine, University of Helsinki, Helsinki, Finland; 4 Addenbrooke’s Hospital, Cambridge University Hospitals NHS Trust, Cambridge, UK; 5 Department of Medicine, University of Cambridge, Cambridge, UK; 6 Department of Pure Mathematics and Mathematical Statistics, University of Cambridge, Cambridge, UK; 7 Department of Haematology, University of Cambridge, Cambridge, UK

**Keywords:** federated learning, machine learning, healthcare, applications, systematic review, privacy, security, methodological advances, deployment, best practices

## Abstract

For healthcare datasets, it is often impossible to combine data samples from multiple sites due to ethical, privacy, or logistical concerns. Federated learning allows for the utilization of powerful machine learning algorithms without requiring the pooling of data. Healthcare data have many simultaneous challenges, such as highly siloed data, class imbalance, missing data, distribution shifts, and non-standardized variables, that require new methodologies to address. Federated learning adds significant methodological complexity to conventional centralized machine learning, requiring distributed optimization, communication between nodes, aggregation of models, and redistribution of models. In this systematic review, we consider all papers on Scopus published between January 2015 and February 2023 that describe new federated learning methodologies for addressing challenges with healthcare data. We reviewed 89 papers meeting these criteria. Significant systemic issues were identified throughout the literature, compromising many methodologies reviewed. We give detailed recommendations to help improve methodology development for federated learning in healthcare.

## The bigger picture

Federated learning (FL) promises to solve the challenges of applying machine learning methods within healthcare, such as isolated datasets, ethical, privacy, and logistical concerns with data sharing, and the lack of diversity in single-center datasets. By connecting multiple sites and keeping data at their source, FL can help address these issues. An increasing number of novel FL methodologies have been developed, and there is an urgent need for the consolidation of this knowledge to address the unique challenges of healthcare data. This review focuses on literature published between 2015 and 2023 that methodologically advances FL for healthcare applications.

## Teaser

Federated learning (FL) is a technique for privacy-preserving machine learning. FL promises to enable large-scale deployment of diagnostic and prognostic tools in healthcare settings. This systematic review of new methodological contributions within FL research highlights innovations in the literature, identifies pitfalls and issues in recent studies, and presents recommendations for future FL practitioners in the development of FL applications in healthcare.

## Introduction

Healthcare data are abundant, representing approximately 30% of the entire global data volume,^1^ and are becoming increasingly available to researchers to allow for such interrogations as trend analysis, pattern recognition, and predictive modeling. This is helped primarily by the increased adoption of electronic health record (EHR) systems in hospitals, with most UK NHS Trusts currently using one and all expected to have one by 2025.^2^ In parallel, there has been a revolution in the capabilities of machine learning (ML) methods, allowing for the efficient analysis of high-dimensional clinical and imaging data.

There are different types and formats of healthcare data, including text from medical notes, imaging data, medical device outputs, wearable signals data, and genomic data. These are usually stored in distinct silos, with EHR data often in a database structure, imaging in a picture archiving and communication system, and medical device/wearable data stored locally. Although there are some well-documented challenges to reproducibility,^3^^,^^4^ ML methods have shown great utility for performing both single and multiple modality modeling of healthcare data.^5^^,^^6^

To create high-quality models that generalize across different data sources, it is most common to pool datasets from different locations and train using the combined dataset (centralized learning). However, this is a serious challenge in the healthcare setting, as there are ethical, privacy, logistical, legal, and security concerns regarding transferring clinical data outside the hospital environment. Additionally, there are logistical issues to ensure data security is maintained in the transfer of such large-scale healthcare data. Finally, each hospital might have its own data transfer and sharing rules, making research across multiple hospitals problematic.

While confidential computing methods, e.g., homomorphic encryption (HE) and secure multi-party computation, are utilized for protecting data privacy during computations, HE alone leads to significant additional computational demands,^7^ and secure multi-party computation faces scalability challenges when handling large datasets.^8^ However, federated learning (FL) incorporates the benefits of secure compute methods while also effectively reducing the computational load and improving scalability. FL also offers a solution to the problems encountered through centralized learning by permitting data to remain locally at each hospital site, with only the ML model being transferred between them. An FL network can be either decentralized or have a central aggregator communicating with all nodes. In the decentralized setting, a model is trained at individual sites. The updated model is passed around to other sites in the network in a defined order, who then initialize from the model, train, and pass it on again. In the centralized aggregator scenario, an ML model is trained at each site, and information about the final model is transferred from all nodes to the aggregator. The aggregator summarizes the model updates from each site and generates a new global model, which is then redistributed to all sites for the local training process to start again. This continues until a pre-defined convergence criterion is met for the global model.

**Figure 1:**
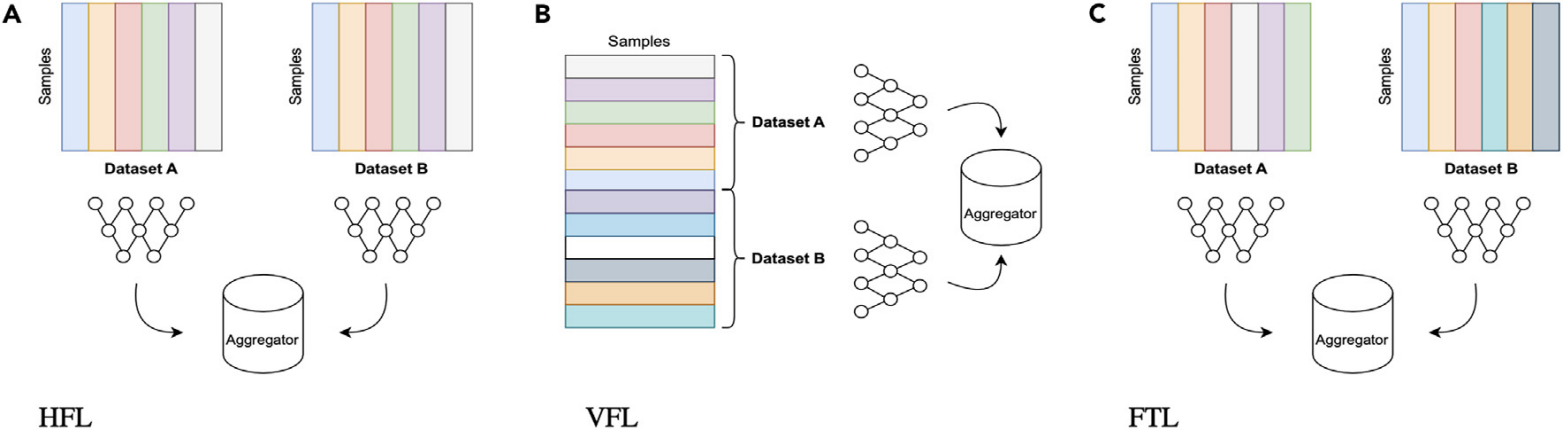
Types of FL Schematic illustrating the feature and sample distributions for horizontal federated learning (HFL), vertical FL (VFL), and federated transfer learning (FTL).

FL methods can be broadly categorized into three groups: horizontal, vertical, and transfer^9^ (see Figure 1). For horizontal FL (HFL) methods, each site holds the same features for different data samples, whereas for vertical FL (VFL), the sites hold different features related to the same samples. For federated transfer learning (FTL), each site has different feature sets that are related to different samples.^9^ Each of these has high relevance for healthcare data, where HFL is akin to learning across different hospitals for common variables and VFL allows for linking of different data silos for, e.g., imaging, EHR, and genomic data. FTL applies most to the real-world clinical environment where different hospitals collect different variables on different people, often dependent on their local clinical protocols.

There are several practical use cases for FL in healthcare including building models for low-prevalence diseases where individual hospitals only have few samples, which may be highly identifiable, while preserving patient privacy. FL also enables cross-institutional collaboration of hospitals in a secured manner, where each member benefits from the access to models trained using data of the other members. Outside of the hospital environment, FL methods can be trained using Internet of Things devices at patients’ homes to collect data and train a model to predict health outcomes.^10^^,^^11^^,^^12^^,^^13^ Finally, FL can be applied in real time for population monitoring, allowing for early disease outbreak detection.^14^ Such a large-scale system can only be achieved through FL, as data from hospitals are often legally prohibited from being shared with other international sites.

With so many diverse application areas, there is now significant literature describing FL methods applied to healthcare challenges such as breast cancer diagnosis,^15^^,^^16^^,^^17^^,^^18^^,^^19^^,^^20^^,^^21^ COVID-19 detection,^22^^,^^23^^,^^24^^,^^25^^,^^26^^,^^27^^,^^28^^,^^29^^,^^30^^,^^31^^,^^32^^,^^33^ length of hospital stay prediction,^34^ and depression diagnosis.^35^^,^^36^ Indeed, in this systematic review, we identified a corpus of 220 such papers between 2015 and 2023.

However, it is also imperative to recognize that healthcare data inherently contain many issues that require special consideration and adjustments to FL methodologies to address. Crucially, in a network of hospitals, each hospital can potentially serve a fundamentally different patient population. This bias can lead to downstream modeling issues if there are, for example, different disease prevalences at different sites or significant differences in the patient numbers at each location.^37^ Each hospital may also follow different clinical practices, leading to differences in data collection and, consequently, issues such as missing data^38^ and non-standardized variable name mappings.^39^ Differences between sites can also manifest in variations in data quality, posing a unique challenge in the best possible aggregation of locally learned parameters.^40^ In addition to the data challenges, privacy is a top concern for all users of healthcare data, and it is critical that FL methods for healthcare are considerate of this when transferring model parameters between the sites or to the aggregator.^41^ Finally, we highlight that hospital environments are typically not equipped with high-performance computational environments, so FL methods must factor in the computational cost of training, transferring, and evaluating models, especially as changes in patient populations or treatment protocols may require retraining of the global FL model and thereby incur additional computational costs.

Therefore, it is surprising that within the corpus of 220 papers, 131 apply existing off-the-shelf FL methods to healthcare datasets, with only 89 papers describing modifications of the FL methodology to address the challenges unique to healthcare data. It is the latter group that this review focuses on, as we are keen to identify which areas of methodology advancement are receiving the focus to address healthcare challenges and whether there are any systematic pitfalls in the way that these new models are being developed.

**Figure 2:**
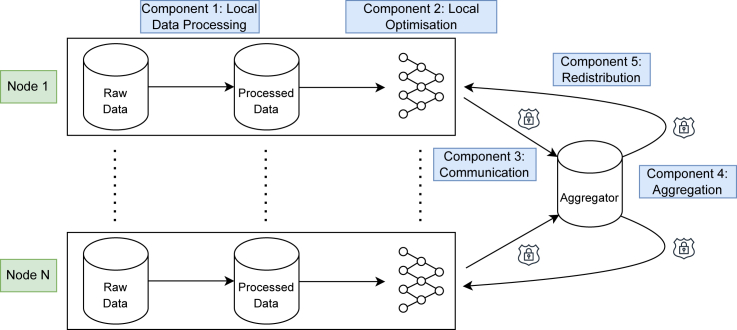
Typical FL workflow The five key components are identified that form the basis of our analysis.

Earlier systematic reviews have considered FL applications in healthcare, including Antunes et al.,^42^ Prayitno et al.,^43^ and Crowson et al.^44^ Our review builds on previous works in several respects. Firstly, this review is much larger than prior reviews, covering 89 papers compared to 44 of the next-largest review.^42^ Secondly, this review has a much larger scope than prior reviews, which focused particularly on EHR data,^42^ data management,^43^ or reproducibility and risk of bias.^44^ Thirdly, we systematically dissect FL into five distinct components (see Figure 2) and highlight the methodological approaches and recent advances in each. Finally, based on our findings, we give direct and practical recommendations (Tables 1 and 2) for each identified challenge such that they might be of immediate use to healthcare practitioners and researchers seeking to use FL in healthcare contexts.

**Figure 3:**
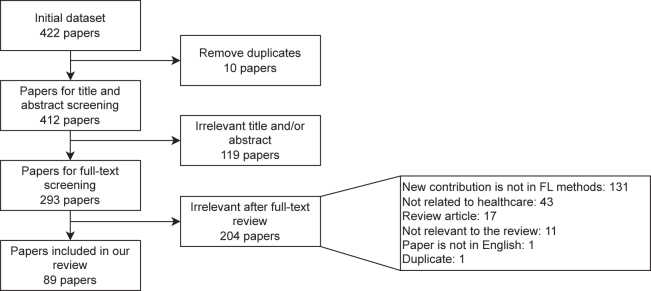
PRISMA flowchart Study selection for our systematic review highlighting the reasons for exclusion of manuscripts at different stages.

**Table 1:** Component-wise recommendations

Component	Recommendations
Local data processing	The types of missingness found in datasets should be detailed and any approaches to imputation stated. Where imaging data are used, extreme care should be taken to ensure that biases in the data are understood and the best practice is followed in the local optimization by using checklists, e.g., CLAIM.^139^ For real-world deployment, it would be preferable for, e.g., feature value normalization to be based on globally exchanged parameters or transformations.^140^ We would encourage authors to consider obscuring their datasets at source before use in the training network, e.g., by a shared hashing function,^115^ noise addition,^141^ or slicing,^142^ to mitigate against data reconstruction attacks. Class imbalances across nodes and within datasets at nodes should be factored into the methodologies, as they are a primary source of bias for FL in healthcare, and it is essential that authors disclose and discuss their strategies to address imbalances, considering potential privacy concerns of data owners. Clinical data can inherently contain large biases and issues from various sources, such as impossible values, missing values, and corruption. Without direct access to the data at each node, automated pipelines should be integrated that can perform a quality and integrity check and remove known sources of bias.^143^ Securely confirming, across nodes, that the demographics are similar and within a tolerance range is also encouraged.^144^ For applying inclusion and exclusion criteria to EHRs or tabular datasets at nodes, authors should disclose the filters used to curate the cohort used in the experiments with, e.g., a structured query language (SQL) query.^145^ We encourage more widespread adoption of internal validation and holdout cohorts to ensure local performance is not overstated due to overfitting of the training data.
Local optimization	It is important for authors to consider whether their solutions are of practical use in the hospital environment, where computing capabilities are more limited than in simulated experiments. The required hardware, in particular whether a GPU is required, should be disclosed in manuscripts and stated as a limitation if not widely available. Outside of FL settings, local optimization is best terminated when the loss converges for a validation cohort of the data. FL, in particular, would benefit significantly from early stopping, as the local node compute times can be highly variable, depending on the sample number and model architecture at each site.
Communication	The cost of sharing data between nodes is a key concern for FL methods in development and deployment. Practitioners must pragmatically balance their concerns around privacy, with the increased computational cost. We therefore give some general advice that may be factored in to the decision-making process. The number of samples at each node may be sensitive and can pose privacy risks if exploited by hostile actors. Sharing this information should be tailored, carefully weighing privacy against utility. Practical strategies include secure sharing through encrypted channels or utilizing aggregation methods that do not require the sample size. In the context of healthcare data, FL networks should use an established cryptography technique^146^ to encrypt as much of the data and communication as possible. It is incredibly important for the aggregator to authenticate and authorize all nodes from which data are accepted and to which data are redistributed. Without this, the network is vulnerable to many different attacks.^147^^,^^148^
Aggregation	Authors should consider whether synchronous or asynchronous updates are preferable for their use case, especially if the local optimizations have very different training times or not all nodes are always available. When the nodes supply updates to the global model, they should be assessed for consistency with other nodes. It should then be investigated if a node shares very different model updates than the others. Of the papers we reviewed, none performed such comparisons.
Redistribution	Communication rounds should be terminated in a principled way. This could be based on the performance of the global model at each node, on a validation or holdout cohort, or the performance of the global model on evaluation data held at the central aggregator.

**Table 2:** Additional recommendations

Component	Recommendations
Deployment	The deployment strategy should consider the entire process, from node distribution to aggregator interaction, ensuring seamless communication and efficient training rounds. Furthermore, the authors should consider integrating machine learning operations (MLOps) practices, as these enhance automation, monitoring, and security; ensure seamless integration and deployment; encourage collaboration; and increase the efficiency and reliability of the FL platform. None of the papers we reviewed discussed version control of the global model. Implementing version control in FL enhances traceability, supports asynchronous communication, enables A/B testing, and provides a rollback mechanism. Storing copies of model artifacts across different versions strengthens auditability and facilitates benchmarking and tracking model performance over time.
Reproducibility	As FL is a rapidly evolving field of innovative research, we recommend that the community work together to develop an FL methodology checklist to improve the documentation of future studies. In the absence of such a checklist, we recommend that authors and reviewers use existing checklists, such as CLAIM,^139^ for assessing the completeness of the data and model descriptions in a medical imaging context. Additionally, tools such as PROBAST^149^ are recommended for assessing the biases in the data and models. Practitioners should only develop a new FL codebase when the existing frameworks fundamentally do not accomplish their aim, otherwise there is a risk of coding errors due to the complexity of the FL system. Codebases, and trained models, should be released publicly if possible to allow the community to easily apply the model and validate the performance.

## Findings in the literature

This section will first give a general overview of the methodological advances in the reviewed studies, followed by a separate analysis of each of the five distinct components of FL depicted in Figure 2. These findings are also summarized in Table S1.

### Study selection

The initial search identified 422 papers that met the search criteria (see Figure 3 and the methods section). After eliminating 10 duplicate papers and filtering to only abstracts and titles focusing on a new approach of FL in healthcare, we retained 293 papers for full-text screening. In this systematic review, we focus on the 89 papers that were relevant to our review question, namely those that introduce a new methodology for applying FL in the context of healthcare.

### Methodology advances

We consider the methodological contributions made to the different FL components identified in Figure 2. The majority of papers (68/89) contribute to a single component, with 18/89 contributing to two and 1/89 contributing to three. Most papers focus on improving the aggregation component (37/89), followed by the communication (35/89) and local optimization (26/89) components. The local data processing component is improved in 5/89 papers and model redistribution in 4/89.

### Types of FL

HFL was the most popular among the three approaches, with 80/89 studies exclusively considering it.^10^^,^^11^^,^^12^^,^^15^^,^^16^^,^^17^^,^^18^^,^^19^^,^^20^^,^^21^^,^^22^^,^^23^^,^^24^^,^^25^^,^^26^^,^^27^^,^^28^^,^^29^^,^^30^^,^^31^^,^^32^^,^^34^^,^^35^^,^^36^^,^^45^^,^^46^^,^^47^^,^^48^^,^^49^^,^^50^^,^^51^^,^^52^^,^^53^^,^^54^^,^^55^^,^^56^^,^^57^^,^^58^^,^^59^^,^^60^^,^^61^^,^^62^^,^^63^^,^^64^^,^^65^^,^^66^^,^^67^^,^^68^^,^^69^^,^^70^^,^^71^^,^^72^^,^^73^^,^^74^^,^^75^^,^^76^^,^^77^^,^^78^^,^^79^^,^^80^^,^^81^^,^^82^^,^^83^^,^^84^^,^^85^^,^^86^^,^^87^^,^^88^^,^^89^^,^^90^^,^^91^^,^^92^^,^^93^^,^^94^^,^^95^^,^^96^^,^^97^^,^^98^^,^^99^^,^^100^ In contrast, VFL^101^^,^^102^^,^^103^^,^^104^^,^^105^ is considered in only five papers and FTL^33^^,^^106^ in four, while two studies^13^^,^^107^ considered both HFL and VFL together.

Data sources are often fragmented across sites. VFL was used when the joining of features across locations was discouraged due to privacy or logistical concerns. For example, when imaging in different modalities was pooled,^102^ different sensors recorded data about the same subject,^104^ or genotype and phenotype data in different sites were linked. FTL was primarily applied to clinical data collected under different protocols where the set of available variables differed between nodes.

### Applications considered

The majority of papers (70/89) applied FL to classification problems; segmentation was addressed in 7/89 reviewed studies, and the remainder focused on problems such as anomaly detection,^78^^,^^93^ tensor factorization,^83^^,^^94^ feature selection,^103^ and regression.^20^^,^^25^^,^^33^^,^^56^^,^^65^^,^^69^^,^^90^ See Table S1 for full details.

### Use of existing frameworks

We find that most papers considered in this review (83/89) develop their own FL framework for FL rather than building on existing platforms such as Flower,^108^ TensorFlow Federated,^109^^,^^110^ PySyft,^111^ FATE,^112^ and NVIDIA FLARE.^113^ Six papers used existing frameworks, namely FATE,^101^ Flower,^32^ NVIDIA FLARE,^99^^,^^102^ and PySyft.^16^^,^^81^^,^^82^

### Public codebases

Only 10/89 studies publicly released their code,^10^^,^^17^^,^^20^^,^^25^^,^^27^^,^^46^^,^^50^^,^^54^^,^^64^^,^^98^ and no papers shared their trained model.

## FL component analysis

We now review each component of the FL pipeline (outlined in Figure 2) as it was applied to healthcare in the 89 considered studies.

### Component 1: Local data processing

#### Methodological advances

In 5/89 papers, the authors focus on improving the data pre-processing. Of these, 4/5 were motivated by problems of class imbalance, with three^12^^,^^56^^,^^79^ using a generative model to create new data samples and one^26^ using augmentation to reduce sample size imbalances across nodes. The remaining paper^91^ used anonymization techniques (such as the quantization of some continuous features) to enhance the privacy of the raw data samples.

#### Datasets

A wide range of data types and sources were considered in the papers reviewed. The most popular sources were imaging data (31/89), sensor data (17/89), and EHR or tabular clinical data (21/89). Other studies considered more niche data sources such as medical devices (7/89), insurance claims data (4/89), and genomic data (2/89). For the imaging studies, a variety of modalities were considered. Chest X-ray (9/31),^22^^,^^23^^,^^26^^,^^27^^,^^28^^,^^29^^,^^30^^,^^31^^,^^32^ retinal (6/31),^31^^,^^56^^,^^62^^,^^63^^,^^66^^,^^70^ microscopy (5/31),^16^^,^^19^^,^^45^^,^^55^^,^^72^ dermoscopy (4/31),^31^^,^^73^^,^^79^^,^^96^ and magnetic resonance (3/31)^71^^,^^77^^,^^102^ imaging constituted the majority. Sensor data were collected from wearable technologies (14/17)^10^^,^^11^^,^^12^^,^^17^^,^^30^^,^^53^^,^^60^^,^^64^^,^^74^^,^^82^^,^^86^^,^^95^^,^^98^^,^^100^ and ambient sensors (4/17).^13^^,^^59^^,^^82^^,^^104^ For papers using EHR or tabular clinical datasets, MIMIC-III^114^ was used in 7/21,^34^^,^^46^^,^^83^^,^^90^^,^^91^^,^^94^^,^^107^ synthetic EHR data in 4/21,^65^^,^^83^^,^^94^^,^^105^ and proprietary data in 2/21.^87^^,^^91^ Offline medical device data were sourced from electrocardiograms (5/7)^51^^,^^57^^,^^97^^,^^101^^,^^106^ and electromyograms (2/7),^59^^,^^86^ while insurance claims data were sourced from the Centers for Medicare & Medicaid Services,^83^^,^^94^ UnitedHealth Group Clinical Research,^25^ and MarketScan Research.^25^^,^^49^ Twelve papers also used popular non-medical datasets, including MNIST (10/12),^23^^,^^35^^,^^45^^,^^63^^,^^64^^,^^76^^,^^79^^,^^80^^,^^88^^,^^95^ CIFAR10 (6/12),^23^^,^^63^^,^^76^^,^^79^^,^^89^^,^^95^ Fashion-MNIST (2/12),^27^^,^^45^ and STL10 (1/12)^23^ in order to benchmark their proposed method’s performance.

#### Outcomes of interest

The scope of the applications considered in the papers was very diverse. A large number (51/89) focused on the diagnosis of diseases such as COVID-19 (12/51),^22^^,^^23^^,^^24^^,^^25^^,^^26^^,^^27^^,^^28^^,^^29^^,^^30^^,^^31^^,^^32^^,^^33^ lung cancer (3/51),^15^^,^^45^^,^^52^ breast cancer (7/51),^15^^,^^16^^,^^17^^,^^18^^,^^19^^,^^20^^,^^21^ skin disease (6/51),^31^^,^^66^^,^^72^^,^^73^^,^^79^^,^^96^ eye disease (4/51),^31^^,^^62^^,^^63^^,^^66^ heart disease (5/51),^85^^,^^97^^,^^101^^,^^105^^,^^106^ brain tumor (4/51),^63^^,^^71^^,^^77^^,^^102^ diabetes (2/51),^56^^,^^70^ neurodegenerative disorders (2/51),^86^^,^^104^ Alzheimer’s disease (2/51),^58^^,^^80^ colorectal carcinoma (2/51),^52^^,^^55^ and sepsis.^54^ There are also applications varying from using bone imaging to predict age^56^ and detecting emotion in speech^67^ to identifying depression through social networking interactions and posts.^35^

#### Data pre-processing

Before training models, data are typically transformed through normalization, transformation, and cleaning. This process was only mentioned in 41/89 papers, performed centrally (before data were allocated to the nodes for synthetic FL experiments) or at a node-by-node level. Commonly used techniques include feature value normalization,^23^^,^^31^^,^^36^^,^^56^^,^^75^^,^^77^^,^^84^^,^^85^ dimensionality reduction,^11^^,^^23^^,^^34^^,^^46^^,^^85^ feature engineering through a data-specific transformation,^11^^,^^12^^,^^13^^,^^23^^,^^53^^,^^89^^,^^91^ and data filtering^11^^,^^30^^,^^49^^,^^54^^,^^61^^,^^75^^,^^77^^,^^84^^,^^85^^,^^107^ for tabular data. For video data, resampling was used,^69^ and for imaging data, resizing,^55^^,^^56^^,^^62^^,^^66^^,^^96^ intensity windowing,^60^ sliding window,^12^^,^^30^^,^^60^^,^^86^ and cropping^19^^,^^48^^,^^56^^,^^77^ were employed.

Most papers do not mention performing quality or integrity checks on the data before or after pre-processing, the exceptions being Gad et al.,^10^ where samples with impossible values are excluded (e.g., negative heart rates) or where inconsistencies in feature values are identified, and Shaik et al.,^11^ who performed principal-component analysis to filter out noise. One paper^105^ examined genotype data for discrepancies and anomalies to ensure reliability and accuracy. Within the 89 papers reviewed, 131 datasets were considered, with the smallest dataset having 116 samples^21^ and the largest having 3.7 million.^91^ None of the papers applied hashing or encryption to the raw data before training.^115^

#### Imbalances in data

There are two potential sources of imbalanced data for FL. Firstly, different nodes in the network can be associated with highly varying sample sizes, and secondly, the prevalence of the outcome variable (class imbalance) can also vary between nodes. In the studies reviewed, imbalances in sample sizes were addressed in only three papers, which employed downsampling^84^ and augmentation.^26^^,^^73^

Imbalance in outcome prevalence was addressed in only four studies. SMOTE^116^ oversampling was used in two papers^12^^,^^22^ to generate a balanced dataset at each node, and deep reinforcement learning was used by Zhang et al.^82^ to encourage devices with balanced class labels to participate more frequently in the local updates. A weighted random forest, assigning higher weights to the less prevalent classes, was used by Gencturk et al.^70^

#### Consistency across nodes

Almost all papers performed their FL experiments in controlled environments without determining whether datasets or their feature values were comparable across the different nodes. The exceptions were Tong et al.,^25^ who computed a surrogate pairwise likelihood function to account for bias between the model parameters from each site and used this to adjust the final model predictions, and Chen et al.,^86^ who used the aggregator to compare each node’s data distribution with a benchmark dataset to estimate the label quality, reflecting the reliability and accuracy of each node’s local data labels. In addition, no papers discussed how the dataset used at each node is curated for use in the experiments, i.e., the systematic collection, organization, and verification of data before the pre-processing stage. Accurate data curation is fundamental in ensuring the reliability and consistency of data cohorts across different nodes in FL systems.

#### Missing data

Most studies did not highlight whether datasets contained any missing values, with only two considering an imputation method while also deleting features with high missingness rates.^46^^,^^104^

### Component 2: Local optimization

#### Methodological advances

In 26/89 papers, the authors focus on improving local optimization. In 6/26 papers, the authors focused on improving the training procedure by using gradient clipping,^102^ modifying the activation function to better tolerate data heterogeneity,^53^ regularizing the model by penalizing deviations from earlier time-series data points,^17^ using contrastive learning for federated model pre-training,^46^ using parameter sharing to reduce model size,^100^ and reordering the data samples to process the most difficult samples at the end of training.^90^ Six papers focus on improving the model architecture to allow for multi-modal data^75^ by tuning the architecture to local datasets,^18^ using smaller binarized neural networks for resource constrained settings,^98^ using extremely randomized trees for privacy preservation,^23^ using an extreme learning machine to directly find the model parameters in one iteration,^104^ and introducing a new method for survival analysis to factor in time-varying covariates.^32^ In 9/26 papers,^12^^,^^20^^,^^27^^,^^57^^,^^66^^,^^72^^,^^89^^,^^96^^,^^107^ the authors decompose the model into parts that are trained on the local nodes and parts trained on the central server. This gives some aspects of the model, which is fine-tuned to local data. In 4/26 papers, the authors design approaches to perform federated semi-supervised learning to utilize unlabeled data samples. In one paper, the authors design a highly stratified cross-validation strategy based on confounding factors to overcome distribution differences between local datasets.

#### Model architectures

In the 82/89 studies that described their model, there was a wide range of complexity, ranging from highly intricate and parameterized models to simpler, traditional ML methods. The most popular choice for model architecture was a convolutional neural network (CNN), with 46/82 studies considering them. Most papers (see Table S1) discuss their own custom CNN architectures (27/46), while many of these papers (19/46) compare several different established architectures such as ResNet (9/46),^16^^,^^27^^,^^29^^,^^48^^,^^56^^,^^62^^,^^63^^,^^72^^,^^96^ DenseNet (3/46),^22^^,^^45^^,^^48^ MobileNet (2/46),^27^^,^^101^ U-Net (46),^32^^,^^66^^,^^71^^,^^102^ AlexNet (4/46),^16^^,^^30^^,^^59^^,^^89^ and LeNet (2/46).^30^^,^^59^ Recurrent neural networks (RNNs) were also popular, with long short-term memory (LSTM) and Bi-LSTM backbones found in 11/82 studies^10^^,^^11^^,^^13^^,^^34^^,^^46^^,^^51^^,^^53^^,^^57^^,^^80^^,^^93^^,^^107^ and five papers using both CNNs and RNNs.^10^^,^^11^^,^^51^^,^^57^^,^^80^ Multi-layer perceptrons (MLPs) were used in 11 studies, with custom architectures for a vanilla MLP employed in 10^18^^,^^20^^,^^21^^,^^24^^,^^55^^,^^67^^,^^74^^,^^75^^,^^84^^,^^87^ and an attention layer incorporated in one.^98^ Some studies also considered FL with more traditional ML algorithms such as gradient-boosted trees,^21^^,^^90^^,^^103^ support vector machines,^21^^,^^85^^,^^91^^,^^103^ fuzzy clustering,^61^ logistic regression,^21^^,^^25^^,^^49^^,^^58^^,^^65^^,^^91^^,^^105^ and random forests.^17^^,^^21^^,^^70^^,^^92^^,^^103^ The remaining studies either used custom algorithms or focused on other aspects of the FL pipeline.

#### Optimizers

Of the 59 papers that mentioned their optimization algorithm, stochastic gradient descent is the most widely used method, found in 35/59 papers, while Adam was used in 20/59 papers (see Table S1). Other methods such as RMSprop,^10^^,^^13^^,^^106^ SAGA,^63^ Adadelta,^26^ maximum likelihood estimation,^65^ or Newton’s method^105^ were also considered. One paper developed their own optimizer.^70^

#### Training initialization

In the 68/89 studies that mentioned the use of initialization strategies for their model parameters, initialization with random weights was the most common (45/67), while 16 papers utilized pre-determined parameters (identified by the authors), 7 used pre-trained weights,^28^^,^^30^^,^^31^^,^^32^^,^^35^^,^^48^^,^^97^ and another used the parameters obtained by the node whose dataset had the highest number of features.^61^ See Table S1 for additional details.

#### Dataset partitioning

Of the papers, 47/89 stated that they partitioned their dataset into internal validation or holdout cohorts. Only two papers^45^^,^^46^ used both an internal and an external holdout cohort. Thirteen papers used an internal validation dataset to avoid model overfitting by using between 5% and 25% of the local dataset.^19^^,^^22^^,^^46^^,^^48^^,^^52^^,^^55^^,^^56^^,^^67^^,^^71^^,^^73^^,^^99^^,^^101^^,^^102^ 15 studies performed 3-,^17^^,^^49^ 5-,^17^^,^^20^^,^^34^^,^^53^^,^^84^^,^^90^^,^^91^^,^^100^^,^^106^ or 10-fold^51^^,^^57^^,^^58^^,^^70^ cross-validation, while two papers^12^^,^^75^ mentioned the use of cross-validation without specifying the number of folds. In 23 papers,^10^^,^^11^^,^^15^^,^^26^^,^^28^^,^^29^^,^^30^^,^^31^^,^^32^^,^^33^^,^^35^^,^^36^^,^^59^^,^^62^^,^^63^^,^^64^^,^^74^^,^^77^^,^^88^^,^^96^^,^^97^^,^^106^^,^^107^ there is a holdout cohort used for model evaluation after a fixed number of rounds of local optimizations. Three papers mentioned the use of test datasets^85^^,^^104^ or validation datasets^86^ but not their sizes.

#### Hardware

Only 40/89 papers mentioned the hardware used in their study. The computational requirements for model optimization varied significantly, and consequently, the hardware used was highly diverse. Most FL methods required either hardware with GPU(s) attached (26/40) or simple CPU machines (15/40). Some studies use edge devices such as a Raspberry Pi^26^^,^^46^^,^^69^^,^^93^^,^^106^ or a smartphone.^59^

#### Privacy-preserving optimization

Six papers^23^^,^^55^^,^^58^^,^^78^^,^^84^^,^^89^ applied differential privacy^117^ during the optimization. This aimed to preserve the privacy of individual samples in the training data. Five papers accomplished this by adding Gaussian noise to the exchanged data, with the sole exception of Hilberger et al.,^55^ who used TensorFlow Privacy^118^ instead.

#### Training termination

Criteria for ending the local optimization are mentioned in 75/89 papers, most of which (67/75) used a fixed number of local training epochs for each node (see Table S1), while four papers^47^^,^^53^^,^^65^^,^^100^ terminated on convergence but did not provide details on how this was determined. Two papers^71^^,^^104^ only required one epoch for training by design.

#### Decentralized training

In seven papers,^18^^,^^31^^,^^36^^,^^53^^,^^60^^,^^74^^,^^93^ the training of the model itself was not entirely performed on the nodes. Three studies trained using both the nodes and edge devices,^18^^,^^31^^,^^93^ and four studies trained small clusters of nodes, which then communicate to the aggregator.^36^^,^^53^^,^^60^^,^^74^

#### Novel developments

One paper removed the need to perform iterations of an algorithm by use of a one-layer MLP, whose weights can be directly solved for,^104^ and another paper partitioned the model architecture and then trained the computationally intensive part on the central aggregator.^89^ Knowledge distillation was used in two studies^10^^,^^48^ where more powerful “teacher” nodes train less powerful “student” nodes. One paper introduced a method where all but the batch normalization layers are exchanged between each node.^19^

### Component 3: Communication

#### Methodological advances

In 35/89 papers, there are contributions to improve the communication component, with five papers even describing contributions to several aspects. Primarily, authors focused on improving encryption methods (11/35), aimed to reduce either the amount of data communicated (12/35) or the number of communication rounds (7/35), and also introduced methods for fully decentralized communication (5/35).^31^^,^^71^^,^^77^^,^^85^^,^^87^ Encryption improvements tended to focus on methods for sharing secret keys among nodes,^54^^,^^88^ encryption mechanisms for the data exchanged,^58^^,^^68^^,^^73^^,^^89^^,^^92^^,^^100^^,^^101^^,^^103^ and also a technique for perturbing model outputs at each node using a secret key.^28^ A reduction in the amount of data communicated is achieved by transferring a subset of the model parameters,^19^^,^^30^^,^^32^^,^^51^^,^^60^^,^^74^^,^^83^^,^^106^ or by compressing, masking and quantizing of gradients or model outputs before exchange.^76^^,^^84^^,^^94^^,^^101^ The number of rounds of communication with the server can be reduced by the inherent design of the model,^25^^,^^34^^,^^56^^,^^65^ by aggregating based on the elapsed time (rather than the epochs),^76^^,^^94^ and by assessing whether a proposed update is beneficial to the network before communicating it.^88^ The remaining papers focus on techniques for detecting attacks during communication^78^ and developing an authentication system for nodes in the network^47^^,^^81^ and systems for client/node management.^64^^,^^73^

#### Data exchanged

In the studies we considered, the data most commonly shared with the aggregator were the model weights (35/89) and gradients (13/89) (see also Table S1). Many papers simply state that they are exchanging “model parameters” (20/89)^13^^,^^19^^,^^20^^,^^23^^,^^30^^,^^33^^,^^34^^,^^46^^,^^47^^,^^52^^,^^53^^,^^61^^,^^62^^,^^64^^,^^66^^,^^67^^,^^78^^,^^84^^,^^85^^,^^99^ or “model updates” (2/89)^22^^,^^86^ without specifically stating if or how these relate to weights or gradients. The outputs of the local models were shared in 7/89^10^^,^^11^^,^^21^^,^^48^^,^^49^^,^^101^^,^^107^ of the papers reviewed. Some papers encrypted the weights before sharing (9/35),^12^^,^^15^^,^^16^^,^^35^^,^^58^^,^^68^^,^^76^^,^^77^^,^^100^ some encrypted the gradients (4/13),^54^^,^^88^^,^^89^^,^^92^ and four papers perturbed the model parameters before sharing.^18^^,^^23^^,^^78^^,^^84^

Beyond the conventional gradient and weight data shared in FL methods, some papers also shared additional information about the data or the training process or procedure. Metadata such as the model architecture, optimizer and loss function,^55^ and training time and maximum performance,^22^^,^^77^ along with the initial learning rate and thresholds used for classification and learning strategy,^51^ were among the extra information exchanged.

#### Encryption

Data were exchanged unencrypted for most of the studies we considered (70/89). Most papers that did encrypt the exchanged data primarily used HE methods (13/19),^12^^,^^15^^,^^16^^,^^17^^,^^54^^,^^68^^,^^73^^,^^76^^,^^89^^,^^92^^,^^100^^,^^101^^,^^103^ while Chen et al.^88^ relied on a symmetric-key algorithm for encrypting the gradients when exchanged. Only three papers^50^^,^^77^^,^^81^ discussed the encryption of the communication channel between the node and the aggregator rather than direct data encryption before exchange. Two papers^35^^,^^58^ stated that they encrypted their data without detailing the method used.

#### Partial model communication

In 12/89 papers, only a subset of the model layers^19^^,^^27^^,^^30^^,^^32^^,^^51^^,^^57^^,^^60^^,^^74^^,^^89^^,^^95^^,^^106^^,^^107^ are communicated. This allows for reduced communication overhead along with improved privacy and more personalized models, as only generic features are exchanged. Other papers accomplish this by sharing only some intermediate model outputs^104^ or randomly shuffling the model outputs.^28^

#### Authentication of nodes

Only two papers^47^^,^^81^ ensured that the node is authenticated as part of the approved FL network before the aggregator accepts the exchanged data. The local model parameters were shared, along with a ring signature, to prove that the data originated from the node.

#### Fully decentralized communication

There were nine papers describing fully decentralized FL methods.^31^^,^^48^^,^^59^^,^^61^^,^^71^^,^^77^^,^^85^^,^^87^^,^^94^ These require communication between nodes rather than a central aggregator, using gradient and weight updates of neighbor nodes to update the local model. Knowledge distillation between the local nodes is employed in two studies^10^^,^^48^ where more powerful nodes behave as teachers to the less powerful student nodes. None of the papers considered the significance of the sequence of updates in the fully decentralized scenario.

#### Optimizing efficiency

Two papers considered approaches to reduce the number of rounds of communication with the aggregator by sharing embeddings of the data with the aggregator (which then trains the model)^104^ and by modeling the pairwise relationships between the data at each node directly,^25^ both of which allow for only a single round of communication. Communication time is optimized in one paper,^31^ where an optimal ring structure between nodes is obtained by solving a version of the traveling salesman problem. Additionally, eight papers focused on reducing the amount of data transferred between the nodes and the aggregator at each iteration. This can be accomplished by sharing only a subset of model parameters^27^^,^^51^^,^^57^^,^^60^^,^^89^ or compressing gradients.^83^^,^^84^^,^^94^ One paper focused on training for several tasks simultaneously to avoid training independent models.^34^

### Component 4: Aggregation

#### Methodological advances

In 37/89 papers, the authors focus on improving the aggregation component. Most commonly, papers consider the weighting of the contributions of the nodes in the network (11/37) by considering the local training loss,^29^^,^^99^ local classification performance,^70^ signal-to-noise ratio of the data,^69^ node similarity,^30^ data quality,^73^ fairness,^45^ Shapley values,^63^ model performance on aggregator test data,^86^ and how each local model performs at all other nodes.^21^

Aggregation methods are often improved by measuring and correcting for the distribution differences between nodes,^25^^,^^33^^,^^65^^,^^105^ allowing for multi-modal data aggregation across different nodes.^13^^,^^101^ In 5/37 papers, the authors cluster nodes together based on their similarity before aggregation, and 5/37 papers focus on improving the aggregation strategy. This is accomplished using hierarchical aggregation,^93^ performing some training rounds on the aggregator,^62^ dynamically selecting participating nodes and scheduling of aggregation,^22^ and the ensembling of the local models to obtain a final model.^49^^,^^102^ The remaining papers focus on methods for asynchronous aggregation (3/37),^35^^,^^59^^,^^97^ using knowledge distillation (2/37),^10^^,^^48^ secure aggregation (2/37),^68^^,^^92^ feature selection in a federated manner,^103^ attack detection during aggregation,^80^ and aggregation of heterogeneous model architectures.^11^

#### Model aggregation techniques

In 78/89 papers, the methods for aggregating the contributions from individual nodes were mentioned. Most papers focused on improving the aggregation strategy, with 10/78 developing custom simple averaging strategies and 31/78 developing customized weighted averaging methods. The well-known FedAvg^119^ aggregation method was used in 14/78 papers, with a modified formulation of it employed in a further 10/78. The remainder of the studies used non-averaging-based methods such as knowledge distillation,^10^^,^^48^ stacking,^11^^,^^49^ split learning,^102^ feature fusion,^101^ federated goal programming,^21^ and training the model one node at a time.^71^ See Table S1 for additional details.

#### Synchronous vs. asynchronous updates

Only four studies^35^^,^^59^^,^^77^^,^^97^ developed FL methods that allowed for asynchronous updates to the global model, while all others required the local nodes to finish their optimization before the updates were applied. Asynchronous updates were made after comparing the performance of the model with the new and previous parameters and only updating the model if the update performed better.

#### Node weighting

A weighting factor was discussed in 50/89 papers to balance the node contributions during aggregation. The majority of papers (38/50) weighted the contribution based on the sample size at each node. Two papers^10^^,^^70^ assigned weights based on the classification accuracy performance of each node, one weighted by considering the credibility of each node^86^ (giving higher weight to nodes whose local model loss on a benchmark dataset lies closer to the global model loss), and another dynamically adjusted the weights based on the variation of loss values from the previous round.^99^ Some papers did not aggregate the local contribution from a node if it was not beneficial to the overall network^35^^,^^88^ or did not satisfy defined performance criteria.^70^ Model training time was considered in one paper^22^ to limit the number of devices transmitting their updates to the aggregator. A reward mechanism was used in one paper^82^ to select optimal nodes, considering the quality of their data and associated energy costs. One paper^49^ compared multiple means from which to determine the weighting during aggregation.

#### Distribution comparisons

In order to address data heterogeneity between sites, only three papers^30^^,^^36^^,^^95^ mentioned that the model distributions between nodes were compared for clustering purposes before aggregation.

### Component 5: Redistribution

#### Methodological advances

In only 4/89 papers, the authors focus on improving the way models are redistributed to local nodes. Two papers personalize the model that is redistributed to the local nodes based on their similarity to other nodes in the network^30^ and by comparing the loss of the global model on a benchmark dataset to the local node losses.^36^ The other two papers focus on improving access to the global model by allowing local nodes or registered third-party researchers to run encrypted inference directly on the global model.^16^^,^^54^

#### Training termination

The criteria for ending model redistribution were specified in 79/89 papers. The majority (69/79) used a pre-determined number of update rounds before termination. Three papers^11^^,^^25^^,^^104^ required only a single round of communication by design. Two papers^97^^,^^105^ used a minimum loss threshold to claim convergence of the global model and terminate redistribution.

## FL evaluation criteria

### Model efficacy

Common ML performance metrics were reported in most papers, such as the model accuracy (59/89), area under the receiver operator characteristic curve (17/89), precision (15/89), F1-score (20/89), sensitivity/recall (18/89), specificity (6/89), Dice score (8/89), and loss value (12/89).

There were 34 papers that benchmarked their proposed methods against other FL methods, while 27 studies compared their simulated FL approaches with classic centralized ML. Given that the focus of this review was on papers introducing novel contributions to the FL methodology, ablation studies were commonly performed to assess the performance impact of including and excluding their proposed modifications.^11^^,^^13^^,^^22^^,^^28^^,^^30^^,^^32^^,^^46^^,^^48^^,^^55^^,^^71^^,^^81^^,^^84^^,^^97^^,^^103^ Seven papers compared the performance of different local model architectures while keeping the FL framework the same.^11^^,^^31^^,^^52^^,^^67^^,^^81^^,^^101^^,^^103^ Furthermore, 34 studies showcase the performance for multiple hyperparameter configurations for their proposed adaptations.

### Communication efficiency

Key metrics for measuring the communication overhead were considered in 18/89 papers, such as communication costs (13/18),^12^^,^^25^^,^^31^^,^^45^^,^^47^^,^^60^^,^^73^^,^^83^^,^^85^^,^^88^^,^^94^^,^^101^^,^^103^ number of communication rounds (4/18),^60^^,^^61^^,^^85^^,^^87^ and the latency (4/18).^17^^,^^22^^,^^59^^,^^101^

### Resource consumption

Time was an important consideration in several papers, including training time (5/89),^18^^,^^59^^,^^76^^,^^103^^,^^104^ model parameter encryption time (2/89),^16^^,^^89^ and authentication signature compute time.^47^ Energy consumption was measured in four papers.^18^^,^^59^^,^^76^^,^^82^ One paper^18^ focused on CPU processing time, total training time, memory usage, and energy consumption for different numbers of offloaded layers in mobile devices.

## Issues identified, recommendations, and remaining challenges

Our systematic review has identified a keen appetite in the research community for developing FL methods with application to diverse healthcare problems. Most studies explore HFL, while VFL is under-explored in the literature. This is surprising, as VFL holds immense potential for addressing healthcare problems, especially considering that healthcare data often have the inherent challenges of being siloed due to logistical, legal, ethical, and privacy concerns related to extensive data linking. In the following, we highlight the many systemic issues in the FL literature that we have identified and give recommendations for corrective action to address them in Tables 1 and 2.

### Datasets: Missing values, modalities, encryption, class imbalances, and partitioning

Careful curation and preparation of data are crucial in ensuring data consistency and quality across nodes; however, we identified many data-related issues throughout the literature. Firstly, given the nature of healthcare data, where different variables will be recorded/available at different sites, it is surprising that no studies discussed structural or informative missingness,^120^^,^^121^ with only two considering the imputation of missing data. One paper^36^ even mentions missing values within their data without describing how the missingness was addressed. It has been shown in other studies that poor quality imputation, and imputation for non-random missingness, can bias a model trained using it.^122^^,^^123^ Only one paper discussed how the authors derived the particular cohort at each node for their analysis. For example, if each node has EHR data, then for each use case, the EHR at each node must be filtered with defined inclusion and exclusion criteria informed through discussions with healthcare professionals. Secondly, we found 32/89 studies applying FL using clinical imaging to resolve the problem that these studies are often limited by low patient numbers due to low disease prevalence at individual sites.^124^ However, given the known challenges of applying ML to imaging data,^125^^,^^126^ FL also has the potential to amplify these issues, as the inherent data biases cannot be explored. Thirdly, where pre-processing of data is used, it is always performed at local nodes or centrally before distribution to the nodes for artificial FL setups. This local approach, however, propagates any biases in feature values through to the pre-processed data. Additionally, while many authors enhanced the network’s privacy by encrypting the exchanged data, all models were trained using pre-processed raw data. No paper considered encrypting data before performing training^115^ or a one-way hashing. This is alarming given that model weights can be highly informative about the raw patient data, whereas hashing of the data at the source allows for breakage of this link.^127^ Fourth, imbalances and distribution shifts are a reality for healthcare data and represent a critical challenge for ML methods. FL methods compound these, as disease prevalence may also vary between nodes.^128^ However, this issue was only considered and addressed in six of the papers we reviewed. We also identified issues in node consistency, a challenge unique to FL. Only one study checked for consistency in the data distributions between sites. In a real-world deployment scenario, biases between different sites are a reality that must be considered.^129^ For example, in an FL network of hospitals, it would be important to understand whether data for pediatric or maternity hospitals were hosted at particular nodes to fully appreciate the age and sex biases inherent to the data. Finally, only two papers employed both an internal validation and a holdout cohort. Most papers considered exclusively either a validation cohort or a holdout cohort. In the wider ML literature, using validation data to mitigate the risks of model overfit and also a holdout cohort for evaluation is standard practice. It is surprising that the FL literature does not echo this.

### Local optimization: Limitations in training and termination

Systemic issues were identified with local optimization methodologies with respect to hardware requirements and training termination. Firstly, in most papers reviewed, the local optimization of models required GPU compute capability attached to the data source, which is not currently found in most hospital environments. There is some progress toward this with the increasing adoption of cloud computing capabilities in clinical environments along with Trusted Research Environments.^130^ However, the lack of widespread computational capability remains a barrier to the mass adoption of FL in real-world healthcare settings. Secondly, most papers train their models for only a fixed number of iterations, with only one mentioning early stopping and two others specifying “until convergence.” This is highly irregular, with principled stopping criteria widespread in the non-FL ML literature, such as early stopping.^131^

### Communication: Metadata, encryption, and authentication

Securely exchanging information among distributed nodes is essential for effective collaborative learning. Nevertheless, this component is fraught with some challenges. Firstly, it becomes apparent from many studies that metadata, beyond the model parameters, were often communicated in parallel to the aggregator. For instance, nearly all papers that relied on FedAvg as an aggregation method required sharing the sample number at each node. This can severely compromise the network’s security if an attacker intercepts the communication or compromises the aggregator by highlighting those nodes that contain a large amount of data to a hostile actor. Secondly, given our focus on healthcare applications, where privacy is a primary concern, it was surprising that most papers developed FL networks that do not encrypt the model parameters when being exchanged with the aggregator. It has been shown that private information contained within the training data can leak into the learned parameters of a model.^132^^,^^133^^,^^134^ Finally, FL networks are susceptible to attacks by a node exchanging inauthentic or false weights or by allowing the network to train on poisoned data.^135^ Only two papers authenticated nodes before accepting their update parameters.

### Aggregation: Central vs. decentralized, synchronous vs. asynchronous, and weighting individual contributions

Effectively combining the knowledge from individual nodes to give an optimal model for a particular use case is the core challenge in FL development. We identified several areas of concern with model aggregation approaches that were systemic to the literature. Firstly, most papers required the use of a central aggregator, with only eight studies exploring a fully decentralized architecture. Decentralization allows for a network that is more robust to failure and attack,^136^ with updates performed mutually between the nodes themselves. Secondly, only three papers discussed a method that allows for asynchronous updates to the global model. Almost all aggregation techniques are simple or a weighted averaging of the contributions from each node. Requiring synchronous updates is a limitation for real-world deployment, as differing local optimization speeds will result in nodes lying idle, waiting for the aggregator to return the global model only once all nodes have finished training. Finally, with real-world data, it is to be expected that nodes may suggest very different updates to the global model. Most papers that disclosed their method simply weighted the contributions by sample size alone. However, it is useful to understand whether those updates are consistent with one another or whether one or more nodes are suggesting updates that are vastly different from the others, as this may be symptomatic of issues with the data or training.^137^^,^^138^ Two papers introduced methods that allow for ignoring or minimizing the contributions of those nodes that are not beneficial to the overall network.

### Redistribution: Termination criteria

Surprisingly, redistribution of the global model was always performed for a fixed number of epochs rather than terminating with a principled stopping criterion. Compared to the wider ML literature, this is aberrant, where early stopping and validation data are used to terminate training.

### Deployment: No real-world setup

It is crucial that real-world deployment is carefully considered and planned for. None of the papers provided evidence about the deployment of their FL platform in a healthcare environment. It needs to be clarified how each node is set up in individual hospitals, how the local model is delivered to these nodes, and how these nodes connect to existing databases. The communication protocols, enabling interactions between nodes and aggregators, were largely unspecified. Moreover, the mechanism or event that triggers new training rounds was not described.

### Reproducibility: Poor documentation and code

Conducting and documenting reproducible research is a cornerstone of the scientific method. However, we identified that the available manuscript documentation and software codebase were mostly not sufficient to allow for the reproduction of the study findings. In particular, we found that key details such as the data pre-processing techniques, data imputation methods, model initialization strategies, and optimizers employed were missing in a large proportion of the papers reviewed. These basic details are crucial to allow for the reproduction of the results that the papers describe. There is also poor documentation of the data exchanged from nodes to the aggregator, with the terms “model parameters” and “model updates” used interchangeably without specifying if these are the gradients, model weights, or some other parameter. It is also very surprising that most papers develop their own implementation of the FL codebase rather than leveraging and building upon existing FL frameworks. The extreme complexity of FL systems leads to concerns that individual implementations are likely to suffer from issues of correctness unless carefully developed.^4^ Also, no papers released trained models, and therefore it is not possible to assess the performance of models independently.

### Limitations of this review

The scope of this review leads it to focus only on those papers whose contribution is to the methodology of FL applied to healthcare. We do not consider manuscripts that apply off-the-shelf FL methods directly to the data. This is a limitation to the review, as many manuscripts have not been considered that claim to have been successfully applied to healthcare data. Additionally, we have not assessed manuscript quality using checklists or performed a review of bias (ROB) assessment. Currently, we have been unable to identify an approach appropriate for FL papers, and it is unclear how to fairly generalize checklists such as CLAIM^139^ or ROB frameworks such as PROBAST^149^ to an FL setting.

### Challenges and future directions

There are numerous challenges to the widespread adoption of FL within the healthcare setting, many of which we have described. We focus on two significant future challenges. Firstly, convergence rates for different FL aggregation methods will continue to be derived, adding to those identified for FedAvg.^150^^,^^151^ Li et al.^150^ have shown that convergence slows significantly if the ratio of the sample numbers between the most prominent and most minor nodes is large. They also demonstrate that the convergence rate of the global model is dependent on the number of local optimization steps. In real-world scenarios, there will be large differences in sample numbers and disease prevalence between the nodes. Deriving aggregation methods that give strong convergence rates of FL, despite these issues, will be a necessary future direction of research. Secondly, the deployment of FL algorithms in healthcare relies on satisfying legal restrictions such as General Data Protection Regulation (GDPR)^152^ and Health Insurance Portability and Accountability Act (HIPAA),^153^ along with regulatory approval for “software as a medical device.” This leads to a potential conflict, as device regulation is expensive and best suited to commercial entities. However, building these FL networks requires strong collaborations and incentivization of hospitals, which is often achieved through academic collaborations. Finally, approaches for dataset curation, pre-processing, and imputation in the federated setting will be further developed along with the widespread adoption of standards and checklists for the development of high-quality FL methods.

## Conclusions

This review focused on the literature describing FL methods for healthcare applications where there were methodological advances. We considered the different areas in which methodological advancements are being made while systematically exploring the application areas and how the FL components were developed. We identified systemic pitfalls in each component and gave recommendations to support practitioners in developing FL methods in healthcare. Specifically, significant improvements are required in areas such as documentation quality, addressing imbalanced and missing data, and sharing non-encrypted updates. The community must also work together to design appropriate checklists for FL methods in healthcare and review bias frameworks for this setting. FL will become a more common and significant tool for healthcare analytics in the future, and by following these best-practice recommendations, we increase the likelihood of adopting these tools in clinical practice.

## Methods

### Review strategy and selection criteria

We performed a search of published works using Scopus for phrases shown in the search terms section covering the period from January 2015 to February 2023. The review was performed using the Covidence^154^ systematic review platform.

#### Search terms

An initial search was performed to extract papers containing one of “federated learning” or “distributed learning” along with one of “classify,” “predict,” “prediction,” “identify,” “predictive,” “prognosticate,” “diagnosis,” “diagnostic,” “diagnose,” “outlier,” “anomaly,” “detect,” or “detecting” in the title or abstract. We also required that “healthcare,” “health,” or “health care” appeared in the abstract. We excluded articles focusing on blockchain development, intrusion detection, remote teaching, and systematic reviews by excluding those papers that included “blockchain,” “block chain,” “classroom,” “class room,” or “attack” in the title or abstract or had “intrusion,” “intrude,” “review,” or “survey” in the title.

#### Title and abstract screening

A team of eight reviewers screened the titles and abstracts for each paper. Each paper was independently assessed by two reviewers, and conflicts were resolved by the consensus of all reviewers.

#### Full-text screening

Nine reviewers performed the full-text screening, with each paper independently assessed by two reviewers. Any conflicts were resolved by the consensus of all reviewers.

#### Data extraction

A team of four extracted the data from each paper used to write the manuscript and assemble Table S1.

## Constoria

The members of the BloodCounts! consortium are Martijn Schut, Folkert Asselbergs, Sujoy Kar, Suthesh Sivapalaratnam, Sophie Williams, Mickey Koh, Yvonne Henskens, Bart de Wit, Umberto D’Alessandro, Bubacarr Bah, Ousman Secka, Parashkev Nachev, Rajeev Gupta, Sara Trompeter, Nancy Boeckx, Christine van Laer, Gordon A. Awandare, Kwabena Sarpong, Lucas Amenga-Etego, Mathie Leers, Mirelle Huijskens, Samuel McDermott, Willem H. Ouwehand, James Rudd, Carola-Bibiane Schӧnlieb, Nicholas Gleadall, and Michael Roberts.

## Supplemental information

Table S1. Data extracted from reviewed studies

Document S2. Consortium authors and affiliations
