# Supplementary material for: Recent methodological advances in federated learning for healthcare
Source: Patterns (N Y). 2024 Jun 14;5(6):101006. doi: 10.1016/j.patter.2024.101006 (PMC11240178; doi:10.1016/j.patter.2024.101006)
Supplement: Document S2. Consortium authors and affiliations [file mmc2.pdf]

Martijn Schut,[9] Folkert Asselbergs,[9] Sujoy Kar,[10] Suthesh Sivapalaratnam,[11] Sophie Williams,[11] Mickey Koh,[12] Yvonne Henskens,[13] Bart de Wit,[13] Umberto D'Alessandro,[14] Bubacarr Bah,[14] Ousman Secka,[14] Parashkev Nachev,[15] Rajeev Gupta,[15] Sara Trompeter,[15] Nancy Boeckx,[16] Christine van Laer,[16] Gordon A. Awandare,[17] Kwabena Sarpong,[17] Lucas Amenga-Etego,[17] Mathie Leers,[18] Mirelle Huijskens,[18] Samuel McDermott,[1] Willem H. Ouwehand,[8] James Rudd,[6] Carola-Bibiane Schönlieb,[1] Nicholas Gleadall,[8] and Michael Roberts[1,6]

[9]Amsterdam University Medical Centre, Amsterdam, Netherlands

[10]Apollo Hospitals, Chennai, India

[11]Barts Health NHS Trust, London, United Kingdom

[12]Health Services Authority, Singapore

[13]Maastricht University Medical Centre, Maastricht, Netherlands

[14]MRC The Gambia Unit, Banjul, The Gambia

[15]University College London Hospitals, London, United Kingdom

[16]University Hospitals Leuven, Leuven, Belgium

[17]West African Centre for Cell Biology of Infectious Pathogens, Accra, Ghana

[18]Zuyderland Medical Center, Zuyderland, Netherlands
